# Supplementary material for: Determinants of the Level of Anti-SARS-CoV-2 IgG ANTibodiEs after Vaccination (DANTE-SIRIO 7) Study in a Large Cohort of Healthcare Workers
Source: Vaccines (Basel). 2022 Dec 12;10(12):2125. doi: 10.3390/vaccines10122125 (PMC9787979; doi:10.3390/vaccines10122125)
Supplement: Supplementary file 1 [file vaccines-10-02125-s001.zip › Figure S1.pdf]

Questionnaire

Name and surname……………………………………………………………………………….

Age…………………………………………...

Personal ID number……………………………..

Phone number………………………………….

Date …………………………………………….

Have you ever been diagnosed with any of the following conditions or have you undergone any of the following medical procedures?

Hypertension	yes	no
Diabetes	yes	no
Coronary artery disease	yes	no
Angina pectoris	yes	no
Myocardial infarction	yes	no
Thromboembolic disease	yes	no
Autoimmune disease	yes	no
Allergy	yes	no
Chronic kidney disease	yes	no
Heart failure	yes	no
Cardiomyopathy	yes	no
Stroke	yes	no
Peripheral arterial disease	yes	no
Chronic obstructive pulmonary disease	yes	no

Family history of premature coronary artery disease (Please mark yes, if any of your parents, brothers, sisters or children were diagnosed with coronary artery disease earlier than at the age 55 for men and 65 for women) yes no

Hyperlipidemia yes no

Do you smoke? Yes No, but I'm an ex-smoker No, I have never smoked

What is your weight?………..kg What is your height………..cm

Date of the second vaccine ………………

Date of the survey………………………...

Number of days between the date of COVID diagnosis and the 1st sampling (3 months from the 2nd dose of the vaccine)………………..

Prior-Covid infection yes no
Positive PCR result on…………..

Course of COVID disease:…………………
1 - HOME INSULATION ONLY
2 - HOSPITALIZATION
3 - HOSPITALIZATION DUE TO COVID COMPLICATIONS

Which of the following symptoms occurred during the COVID disease? (you can choose several answers)

Lack of any symptoms	yes	no
Malaise	yes	no
Loss of smell	yes	no
Loss of taste	yes	no
Feverish state (<38°C)	yes	no
Fever (≥38°C)	yes	no
Runny nose	yes	no
Cough	yes	no
Sore throat	yes	no
Shortness of breath	yes	no
Respiratory failure requiring oxygen therapy	yes	no
Muscle pain	yes	no
Gastrointestinal complaints	yes	no

Headache	yes	no
Others	yes	no

Which of the following symptoms occurred after 1st dose of vaccine? (you can choose several answers)

Lack of any symptoms	yes	no
Injection site soreness	yes	no
Malaise	yes	no
Loss of smell	yes	no
Loss of taste	yes	no
Feverish state (<38°C)	yes	no
Fever (≥38°C)	yes	no
Runny nose	yes	no
Cough	yes	no
Sore throat	yes	no
Shortness of breath	yes	no
Respiratory failure requiring oxygen therapy or ventilator	yes	no
Muscle pain	yes	no
Gastrointestinal complaints	yes	no
Headache	yes	no
Others	yes	no

Which of the following symptoms occurred after 2nd dose of vaccine? (you can choose several answers)

Lack of any symptoms	yes	no
Injection site soreness	yes	no

Malaise	yes	no
Loss of smell	yes	no
Loss of taste	yes	no
Feverish state (<38°C)	yes	no
Fever (≥38°C)	yes	no
Runny nose	yes	no
Cough	yes	no
Sore throat	yes	no
Shortness of breath	yes	no
Respiratory failure requiring oxygen therapy or ventilator	yes	no
Muscle pain	yes	no
Gastrointestinal complaints	yes	no
Headache	yes	no
Others	yes	no

Do you take any medications yes no

which may reduce immunity?

If yes, please provide a name…..
