# Supplementary material for: Determinants of the Level of Anti-SARS-CoV-2 IgG ANTibodiEs after Vaccination (DANTE-SIRIO 7) Study in a Large Cohort of Healthcare Workers
Source: Vaccines (Basel). 2022 Dec 12;10(12):2125. doi: 10.3390/vaccines10122125 (PMC9787979; doi:10.3390/vaccines10122125)
Supplement: Supplementary file 1 [file vaccines-10-02125-s001.zip › Table S1.pdf]

Table S1

Multivariable linear regression model for significant determinants of SARS-CoV2-IgG after full vaccination with BNT162b2.

	Value	Std.Error	p-value
(Intercept)	2220.30033	119.90045	0.0001
Prior COVID infection	959.58909	117.16105	<0.0001
Time of sampling	-1001.42744	63.20564	<0.0001
Fever after 1st dose	1046.29586	275.07431	0.0002
Muscle pain after 1st dose	297.39899	147.46381	0.044
Lack of symptoms after 2nd dose	-279.91261	140.53405	0.046
Fever after 2nd dose	611.47676	153.58695	<0.0001

R2	upper.CL	lower.CL
0.198	0.237	0.166

.
